# Connecting genes, coexpression modules, and molecular signatures to environmental stress phenotypes in plants

**DOI:** 10.1186/1752-0509-2-16

**Published:** 2008-02-04

**Authors:** David J Weston, Lee E Gunter, Alistair Rogers, Stan D Wullschleger

**Affiliations:** 1 Environmental Sciences Division, Oak Ridge National Laboratory, Oak Ridge, Tennessee 37831-6422, USA; 2 Environmental Sciences Department, Brookhaven National Laboratory, Upton, NY 11973-5000, USA; 3 Department of Crop Sciences, University of Illinois at Urbana Champaign, Urbana, IL 61801, USA

## Abstract

**Background:**

One of the eminent opportunities afforded by modern genomic technologies is the potential to provide a mechanistic understanding of the processes by which genetic change translates to phenotypic variation and the resultant appearance of distinct physiological traits. Indeed much progress has been made in this area, particularly in biomedicine where functional genomic information can be used to determine the physiological state (e.g., diagnosis) and predict phenotypic outcome (e.g., patient survival). Ecology currently lacks an analogous approach where genomic information can be used to diagnose the presence of a given physiological state (e.g., stress response) and then predict likely phenotypic outcomes (e.g., stress duration and tolerance, fitness).

**Results:**

Here, we demonstrate that a compendium of genomic signatures can be used to classify the plant abiotic stress phenotype in *Arabidopsis *according to the architecture of the transcriptome, and then be linked with gene coexpression network analysis to determine the underlying genes governing the phenotypic response. Using this approach, we confirm the existence of known stress responsive pathways and marker genes, report a common abiotic stress responsive transcriptome and relate phenotypic classification to stress duration.

**Conclusion:**

Linking genomic signatures to gene coexpression analysis provides a unique method of relating an observed plant phenotype to changes in gene expression that underlie that phenotype. Such information is critical to current and future investigations in plant biology and, in particular, to evolutionary ecology, where a mechanistic understanding of adaptive physiological responses to abiotic stress can provide researchers with a tool of great predictive value in understanding species and population level adaptation to climate change.

## Background

The advent of high-throughput genome sequencing coupled with breakthroughs in the field of functional genomics has provided an unprecedented opportunity to study the molecular mechanisms that govern the dynamic behavior of cells, organs, and organisms [1]. Indeed, there are excellent examples documenting interdisciplinary use of these emerging technologies, from human genome SNP scans diagnostic of human disease susceptibility [2,3] to discovery of the genetic mechanisms underlying beak morphology of Darwin's finches [4]. Applications are also apparent in plant biology, where the use of genomic technologies have uncovered stress-dependent behaviors in mechanistic detail (see [5] for a review). Such studies have led to the elucidation of highly complex and interacting networks of the abiotic stress response. For example, salinity, drought, and cold elicit a dehydration response that shares many common elements and interacting pathways [6,7]. These findings have spurred additional investigations searching for shared signaling cascades or molecules associated with pathway integration, or cross-talk, and have led to numerous candidates including reactive oxygen species (ROS) and calcium signaling [8,9], hormones [10,11] and others [12-14]. However, despite the advances made possible by "omics"-based technologies, we still struggle to accurately associate the genes, transcriptional cascades, and signaling networks with physiological performance and ecological fitness.

One obstacle to this lack of association is perhaps the result of two opposing paradigms often used in comparative physiology [15]. The first approach, termed gene-to-phenotype, is typified by that of many "omics"-based studies where the effects of specific genes on phenotypic performance and fitness are evaluated (e.g., a reverse genetics approach, [16]). This is in contrast to the phenotype-to-gene approach where the biologist attempts to determine the evolutionary potential of a given trait within a population without identifying the underlying genes (e.g., ecological genetics [17]). Thus, the latter approach is interested in the potential for a trait to evolve, while the former focuses on the underlying genetic mechanism of a particular trait. The integration of both approaches will be an important component of the emerging field of evolutionary and ecological genomics, which aims to study adaptation of natural populations to their environment [18].

To fully understand the genetic mechanisms underlying physiological adaptation to abiotic stress, we must first begin to understand the complex biological processes of how the resultant phenotype is generated from the genotype and then seamlessly coalesce our newfound understanding with population and evolutionary genetics. To initiate this task, we have adapted and integrated two recent analytical advances from the biomedical community. The first approach uses a novel weighted gene coexpression network to determine signaling networks and core genes underlying disease states and evolutionary diversification [19-21]. The second approach explores the genomic signature concept as recently defined by Lamb et al. [22], and is currently used to connect the disease state of an organism with the underlying genes and possible drug treatments [23]. Our purpose is to determine if these techniques can be used to associate the abiotic plant stress transcriptome with common and specific pathways underlying phenotypic response in a manner that is conducive to current and future genetic studies. We address this by combining gene coexpression networks with the genomic signature concept to investigate transcript profiles for plants exposed to drought, osmotic, salt, cold, heat, and UV-B stress. Our intent is not to describe in exhaustive detail the genes unique to or common among these stresses, although we do this to some extent, but rather to illustrate the power of this approach and provide sufficient information so that we and others can evaluate the full potential of this technique for plant biologists and evolutionary ecologists.

## Results

### Arabidopsis stress gene coexpression network

It is known that the plant stress response is characteristic of highly complex and often integrated signaling pathways [6-12]. To help elucidate the transcriptional networks associated with exposure to abiotic environmental stress, a weighted gene coexpression network was constructed as described in Zhang and Horvath [20] and in Materials and Methods from a subset of the AtGenExpress abiotic stress dataset [24]. The data subsets were determined by first analyzing all abiotic stress datasets separately for differential gene expression between control and treatment conditions using the limma package [25] operated within the R statistical program language [26]. Genes that had an adjusted *p *< 0.01 and a log-odds ratio > 1.5 were deemed significantly differentially expressed and were subsequently included in the data subset. This subset, or initial input gene list, contained 16,036 (~57% of the genome) unique gene transcripts identifiers with significantly higher or lower abundance at least once per treatment and per time-point [see Additional file 1].

**Figure 1:**
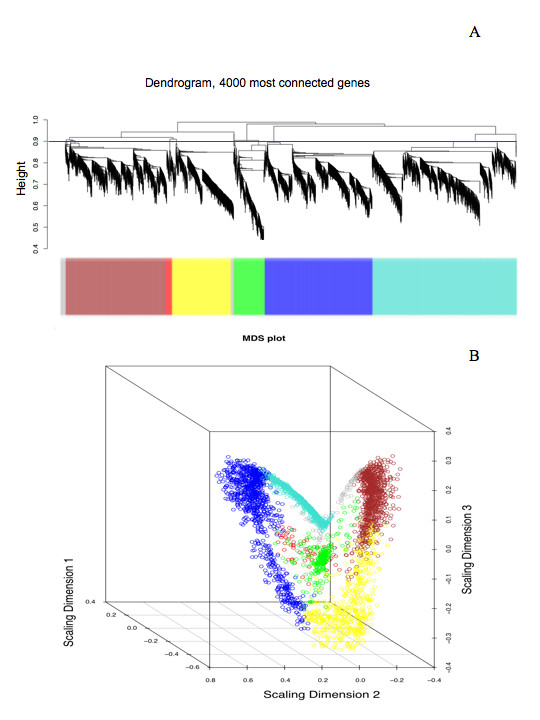
**Visual representation of the AtGenExpress abiotic gene coexpression network**. (a) A dendrogram of the 4000 most connected genes grouped into six distinct coexpression modules. The red line indicates the height at which the tree was cut to produce the distinct gene clusters (modules) as denoted by the color bar. (b) Multi-dimensional scaling plot of the gene coexpression network. Each circle represents a single gene and the color of the circle corresponds to module designation. The distance between circles is a function of the topological overlap and provides a visual representation of gene and module relationships within the network.

The network construction algorithms were applied to normalized raw intensity transcript abundance values across all microarray samples (*n *= 64) for designated genes from the above subset list. Due to computational constraints, only the ~66^th ^quantile (4000) of the most highly connected nodes (genes) were subjected to unsupervised hierarchical clustering to define groups of highly correlated gene expression patterns, termed modules. Using the above criteria, six unique modules were found to have high expression similarity (connectivity), and were subsequently assigned individual colors (Fig. 1).

**Figure 2:**
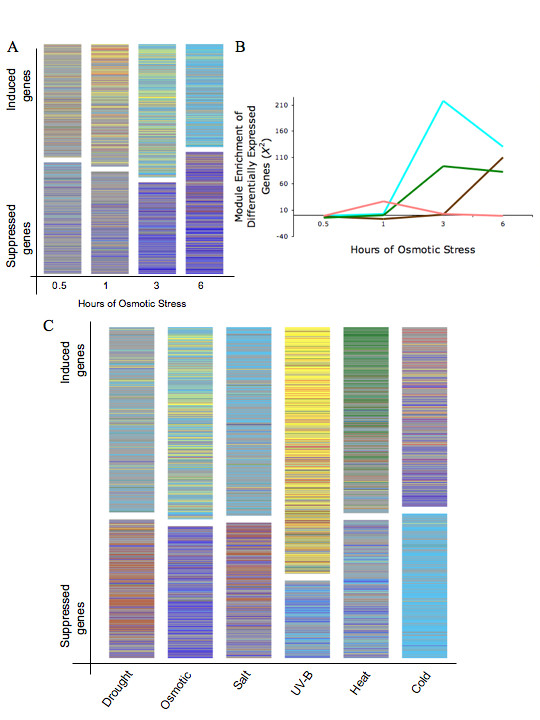
**Relationship between expression abundance and module association**. (a) Patterning of gene expression architecture relative to time of osmotic treatment. The patterns are structured according to expression abundance and the corresponding gene module. (b) Relationship between Chi square enrichment (*X*^2^) of differentially expressed genes and time of osmotic stress treatment. (c) Visual patterning of gene expression architecture relative to 3 h of drought, osmotic, salt, UV-B, heat, and cold treatments.

To determine the relationship between module designation and environmentally-induced expression patterns, we rank ordered all genes according to the log_2 _fold-change in transcript abundance between treatment and control and then color-coded each gene according to its corresponding module color. The resultant ranked gene-lists showed clear patterns in response to treatment duration (Fig. 2A & Fig. 2B). In the osmotic condition, for example, yellow (Pearson *X*^2 ^test, *p *< 2.0 × 10^-6^) and red module (*p *= 7.0 × 10^-7^) genes significantly enriched the distribution of up-regulated genes at the initial time point, with both modules maintaining significantly enriched distributions throughout the treatment period [see Additional file 2]. However, at three hours of osmotic treatment, blue module genes began to enrich down-regulated genes (*p *= 1.6 × 10^-5^), while turquoise module genes enriched up-regulated genes (*p *= 3.0 × 10^-4^). Temporal trends for module enrichment of up- and down-regulated genes were apparent for all treatment conditions (Fig. 2C), and are summarized [see Additional file 2].

**Figure 3:**
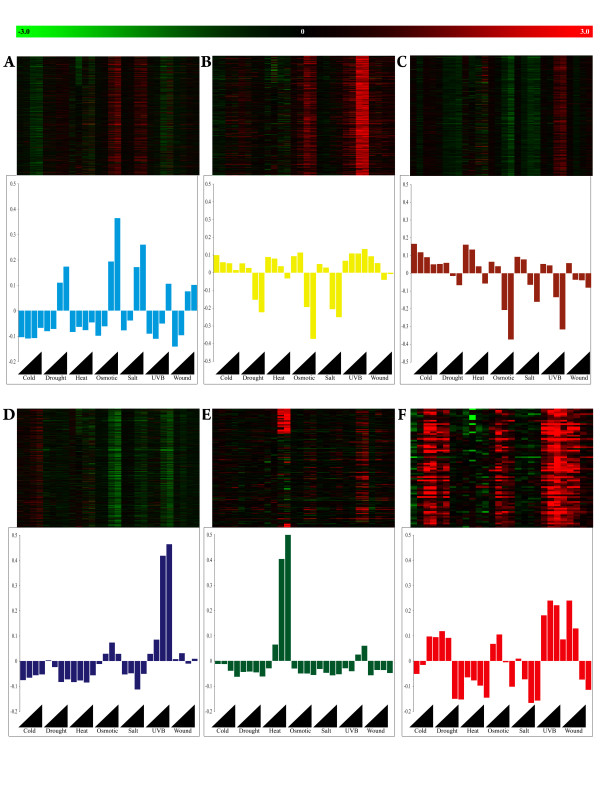
**Relationship between modules and plant stress phenotype**. Red-green heat maps depict mean differential gene expression between control and treatment conditions (x-axis) for all genes specific to the turquoise (A), yellow (B), brown (C), blue (D), green (E), and red (F) modules. Each horizontal line within a heat map shows the expression values (in terms of color) for the same gene across treatments. Red is increased expression, black is neutral, and green is decreased expression in comparison to the control treatment. The black triangles denote the direction of increasing treatment exposure. The corresponding bar plots are the eigengene values, first principle component, as determined from singular value composition for each module. Each bar is the average of two eigengene values.

According to network theory [27,28], genes within coexpression modules often share conserved biological functions. To investigate the functional relationship between individual modules and stress response, we used the singular value decomposition method as developed by Alter et al. [29] and applied by Oldham et al. and Horvath et al. [19,30]. This method characterizes the expression of each module by its first principle component- eigengene value. In general, the results from the singular value decomposition corroborated the visual patterns from the rank lists (Fig. 2) and distribution of module genes within differentially expressed genes [see Additional file 2]. For example, the greatest eigengene value for the green module was for the heat treatment (Fig. 3E; *p *= 5.4 × 10^-5^, Kruskal-Wallis test). Using the GOStat program for gene ontology analysis (, [31]), the most overrepresented GO category for the green module was in response to temperature stimulus (GO:0009408, *p *= 4.4 × 10^-22^).

The green module contained genes known to participate in the heat response pathway including many of the heat shock proteins (HSP 83- At5g52640, HSP23.5-At5g51440, HSP17.6-At5g12030, mtHSP70-At5g09590, HSP21-At4g27670, mtHSP23.6-At4g25200, HSP22-At4g10250, HSP17.4-At3g46230, HSP70-At2g32120, HSP101-At1g74310, HSP-17.6-At1g53540). In addition, the green module eigengene significantly correlated with the expression of heat responsive markers genes At5g59720 (Pearson cor = 0.95, *p *= 10 × 10^-20^) and At4g36990 (Pearson cor = 0.78, *p *= 4.6 × 10^-14^) as previously reported [32-34].

The brown module showed a slight yet significant relationship to the cold treatment (Fig. 3C; *p *= 1.3 × 10^-3^, Kruskal-Wallis test) and was enriched with gene products targeting the thylakoid (GO:0044436, *p *= 1.4 × 10^-78^) and that participate in photosynthesis (GO:0015979, *p *= 1.8 × 10^-7^). The module was most associated with the early stages of cold stress as determined by differential gene enrichment [see Additional file 2] and the -0.57 Pearson correlation (*p *= 10 × 10^-20^) between eigengene value and the known cold responsive marker genes [33,35,36] COR15A (At2g42540), but not in At4g25480 (DREB1A), as previously reported [32].

The turquoise module showed a significant relationship for both salt (Fig. 3A; *p *= 5.4 × 10^-5^, Kruskal-Wallis test) and osmotic (*p *= 5.4 × 10^-5^) treatments. The module is enriched with GO categories in starch metabolism (GO:0005982, *p *= 1.9 × 10^-5^), protein/peptide degradation (GO:0009056, *p *= 1.1 × 10^-4^), and included dehydration responsive genes (At5g66400-RAB19, AT5G52300-RD29B, At5g25610-RD22, At3g56080, At1g01250-DREB A4), early dehydration responsive genes (At1g08930-ERD6, At1g69450-ERD4 like) and late embryogenesis abundant genes (At5g06760, At4g36600, At4g13560, At2g35300, At1g32560). The module eigengene value significantly correlated with known maker genes for salt [33,37,38] (ATPLC1, Pearson cor = 0.68, *p *= 7 × 10^-10^) and osmotic stress (KIN1, cor = 0.74 *p *= 3.5 × 10^-12^; COR78, cor = 0.64, *p *= 1 × 10^-8^) [33,39,40], further suggesting a role for the turquoise module in the dehydration responsive program.

The blue module showed a significant relationship with the UV-B treatment (Fig. 3D; *p *= 2.2 × 10^-5^, Kruskal-Wallis test), which is characterized as a down-regulation response. According to ontology analysis, blue module genes tend to encode products that are involved in protein modification (GO:0006464, *p *= 1.3 × 10^-19^) including amino acid phosphorylation (GO:0006468, *p *= 3.2 × 10^-25^) and translation (GO:0043687, *p *= 9.3 × 10^-21^).

The yellow module also showed a significant relationship with the UV-B treatment (Fig. 3B; *p *= 2.2 × 10^-5^, Kruskal-Wallis test), except that the module genes were up-regulated. Ontology analysis indicated that the yellow module was enriched for macromolecule biosynthetic processes (GO:0009059, *p *= 1.3 × 10^-90^) including organelle biogenesis (GO:0006996, *p *= 4.8 × 10^-64^) and ribosome biosynthesis and assembly (GO:0042254, *p *= 6.8 × 10^-58^). The blue module showed a significant relationship with the UV-B marker At2g24850 (Pearson cor = 0.7, *p *= 1.3 × 10^-10^), but not At5g52250 (cor = 0.05, *p *= 0.67). Alternatively, the yellow module displayed a weak yet significant relationship with At5g52250 (cor = 0.26, *p *= 0.037), but not At2g24850 (cor = -0.13, *p *= 0.29) as previously reported [32].

According to the singular decomposition values and differential gene enrichment analysis, the red module showed significant relationships with nearly all stress treatments [see Additional file 2, Fig. 2]. Ontology analysis indicted that the red module was overrepresented with genes participating in signal transducer activity, including transmembrane receptor activity (GO:0004888, *p *= 7.6 × 10^-3^) as well as response to environmental stimuli (GO:0050896, *p *= 0.013). Interestingly, the most connected gene within the red module, or hub, is an uncharacterized ankyrin repeat family protein [At5g54720; see Additional file 3], which has been shown to regulate salicylic acid signaling. Genes involved in calcium-based signaling also enriched the red module, including calcium dependent protein kinases, calmodulin related proteins, calcium and calmodulin binding proteins [see Additional file 3].

### Genomic signatures

As illustrated above, genes with significantly higher or lower transcript abundance were associated with specific modules depending on the duration of and kind of stress treatment. To relate patterns of genome-wide mRNA expression to phenotypic state, we adapted the genomic signature concept from Lamb et al. [22] where statistical approaches are used to scan an unknown query signature against a database of known reference profiles. For our purposes, the database of reference profiles was created from the above AtGenExpress dataset specific to UV-B, heat, salt, cold, osmotic, and drought treatments at the four initial time-points already detailed above. The 'query' signatures were derived from independent studies imposing UV-B [41] and cold [42] treatments as well as our own heat and drought investigations. In contrast to the Lamb et al. [22] approach, we used ordered list statistics [43-45] to determine structural similarities among gene ranks of query and reference signatures.

**Figure 4:**
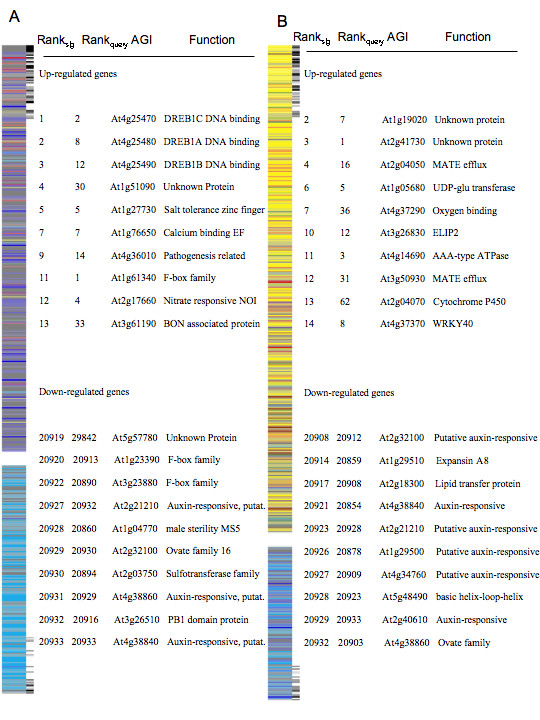
**Gene expression signature comparisons**. (a) Visual display of the 3 h reference cold signature and identification of similarly ranked genes, as denoted by adjacent black and gray lines, with the query cold signature. The rank, identification, and annotation for the 10 most similarly ranked up- and down-regulated genes are described. (b) Display of the 6 h UV-B reference signature and identification of similarly ranked genes with UV-B query signature. The rank, identification, and annotation for the 10 most similarly ranked up- and down-regulated genes are described.

To investigate the integrity of the reference database and the concept in general, we generated a rank-based signature from an independent expression profiling experiment conducted by Lee et al. [42]. The authors imposed a 0°C cold treatment starting at 12 PM under light for 0 (untreated control), 3, 6, and 24 h on two-week old *Arabidopsis *seedlings grown in agar medium. Query signatures from the 3 and 6 h time-points had the highest similarity scores against the 6 h cold reference, 380 and 533, respectively (Table 1, Fig. 4A). The intersect between query and reference signatures (genes driving the similarity score, see Material and Methods) consisted of known dehydration responsive marker genes, namely DREB family genes, including DREB-1A (At4g25480), DREB-2A (At5g05410), DREB-1C (At4g25470), and DREB 1B (At4g25490), [see Additional file 4]. The 24 h cold query list showed similarity to the 6 h cold reference signature (similarity = 157), but also had similarity to the 3 h osmotic (similarity = 218) and the beginning stage of the drought reference signature (0.5 h drought, similarity = 148). Previous research has shown a close link between dehydration and cold responsive signaling in the past and our results support this notion [6,7].

**Table 1:** Signature comparisons. Similarity score comparisons for independently derived query signatures for cold at 3 h, 6 h, and 24 h [42]; UV-B [41]; heat alone, drought alone, and heat and drought applied simultaneously; scanned against the reference signature database. The most similar scores per treatment are bolded. The top row represents time in (h) for stress treatment duration of the reference signature.

	**Cold (h)**				**Drought(h)**				**Heat(h)**				**Osmotic(h)**				**Salt(h)**				**UV-B(h)**			
	**0.5**	**1**	**3**	**6**	**0.5**	**1**	**3**	**6**	**0.25**	**0.5**	**1**	**3**	**0.5**	**1**	**3**	**6**	**0.5**	**1**	**3**	**6**	**0.5**	**1**	**3**	**6**
**Cold 3 h**	63	89	96	**380**	89	176	171	73	11	4	37	42	109	166	37	47	36	10	10	4	110	180	63	29
**Cold 6 h**	38	148	159	**533**	169	173	159	24	1.5	8	8	1	116	153	154	82	14	35	35	36	199	198	110	42
****Cold 24 h****	25	54	54	157	**148**	76	76	35	8.4	17	20	20	41	56	**218**	160	16	50	50	139	123	94	63	57
**UV-B**	22	10	11	75	66	45	46	30	3	32	20	21	*69*	131	219	175	13	21	21	89	168	181	**458**	380
**Heat singular**	3	14	18	49	17	35	32	139	10	108	192	196	27	24	48	88	49	13	13	112	3	3	30	108
**Drought singular**	12	10	12	87	79	67	63	**183**	1	16	4	4	96	53	**219**	191	*9*	11	11	164	62	73	157	146
**Heat & Drought Simultaneous**	5	28	30	78	29	44	45	**127**	11	116	181	**186**	40	26	62	96	48	8	8	103	15	8	52	117

Next an independent UV-B specific query signature was generated from Brown et al. [41] data to scan against our reference database. Despite differences in growth and treatment induction conditions, the query signature showed strong similarity with the UV-B 3 h and 6 h reference signatures (Table 1, Fig. 4B). Interestingly, the common intersect genes between reference and query signatures were enriched with transcription factors, particularly the WKRY family At1g80840, At2g38470, At5g24110 and zinc finger family At1g27730, At5g27420, At5g27420, At5g59820, [see Additional file 4].

**Figure 5:**
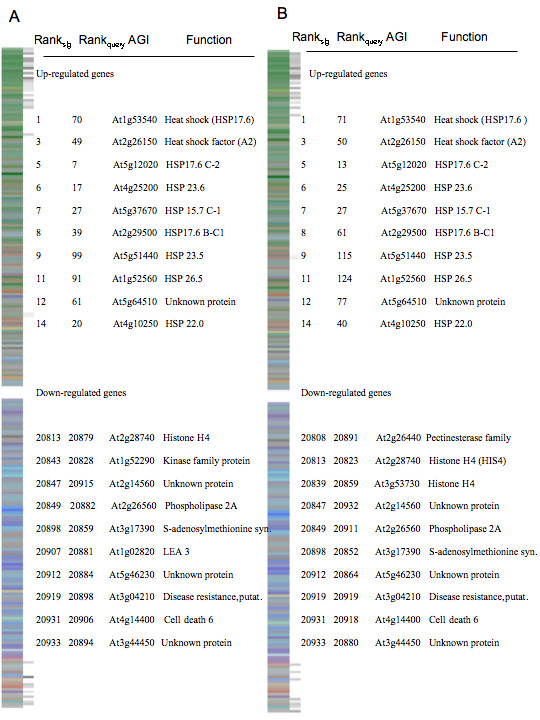
**Gene expression signature comparisons**. (a) Visual display of the 3 h reference heat signature and identification of similarly ranked genes, as denoted by black lines, with the singular query heat signature. The Rank, identification, and annotation for the 10 most similarly ranked up and down genes are described. (b) Display of the 3 h heat reference signature and identification of similarly ranked genes with simultaneously imposed heat and drought query signature. The Rank, identification, and annotation for the 10 most similarly ranked up and down genes are described.

**Figure 6:**
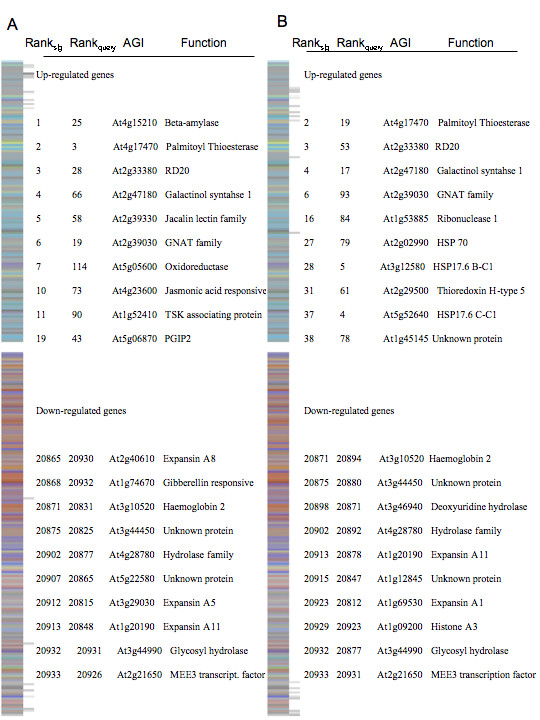
**Gene expression signature comparisons**. (a) Visual display of the 3 h reference drought signature and identification of similarly ranked genes, as denoted by black lines, with the singular query drought signature. The Rank, identification, and annotation for the 10 most similarly ranked up and down genes are described. (b) Display of the 3 h drought reference signature and identification of similarly ranked genes with simultaneously imposed heat and drought query signature. The Rank, identification, and annotation for the 10 most similarly ranked up and down genes are described.

Under field conditions, plants are often exposed to multiple environmental conditions that impact yield and fitness [46]. To test if the signature concept could be applied to multiple stress treatment conditions, we conducted an expression profiling experiment on plants exposed to heat, drought, and then heat and drought in combination (refer to Materials and Methods). The singular heat stress scan yielded high similarity scores specific to heat at 1 h and 3 h, with similarity scores 192 and 196, respectively (Table 1, Fig. 5A). Intersect genes common to query and heat reference signatures contained genes known to participate in heat responsive pathways, particularly the heat shock proteins (mt-HSP At4g25200; sm-HSP At2g29500, sm-HSP At2g19310, HSP 17.6 At1g53540). The scan from the singular drought treatment showed high similarity to dehydration responsive signatures including drought at 6 h and osmotic 3 h and 6 h references (Table 1, Fig. 6A). Nine genes were common to query signature, and drought and osmotic reference signatures [see Additional file 4] including a water responsive transcription factor (At1g52890), salt stress responsive gene (RD20, At2g33380), and fungal defense response (respiratory burst) (At5g64120).

We next created a signature from the simultaneously imposed heat and drought treatments to query the reference database. Interestingly, the two highest similarity scores were 3 h heat (Table 1, Fig. 5B) and 6 h drought (Table 1, Fig. 6B), indicating that the genomic signature concept as applied here has the potential to detect more than one environmental perturbation within a single treatment. Unfortunately, the significance of the similarity score is difficult to interpret. To address this more specifically, we decomposed the dual stress signature into its 6 independent replicates [see Additional file 5]. The results still place drought and heat as the two most similar signatures yet the significance of the drought similarity is weak. However, the similarity score becomes significant when the depth of the signatures is increased [see Additional file 5 for further discussion]. Nonetheless, the query signature had 17 genes in common with the drought signature including the drought responsive marker RD20 (At2g33380), and contained 26 genes in common with the heat reference including many of the heat shock protein encoding genes and a number of genes encoding DNA heat shock N-terminal domain-containing proteins [see Additional file 4].

## Discussion

The goal of our study was to demonstrate the use of an integrative systems approach for connecting gene expression patterns to physiological characteristics, thereby providing mechanistic insight into genome function under abiotic stress conditions. Central to our approach is the use of the genomic signature concept to characterize the plant stress phenotype and provide a link to the underlying network pathways, modules, and eventually genes. The use of expression array data to create a signature cataloging system (reference signature database) has been used previously to characterize chemical perturbations on tissue samples and cell culture populations [47,48], and more recently to link genes and disease states to potential therapies [22,23]. In the present study, we extend the signature cataloging approach to plant biology/ecological genomics by using the ATGenExpress abiotic stress dataset to compile our first-generation reference signatures database.

Validation of the reference database, and the approach in general, was accomplished with independent datasets for UV-B [41], cold [42] and our own datasets for heat, drought, and the simultaneously imposed heat-drought treatments. Altogether, more than half of the stress treatments included in the signature database were scanned by independent query signatures. Our results are encouraging and show that despite differences in array platform, growth conditions, and even the application of treatments, the signature approach is robust in classifying the plant stress phenotype. This was particularly evident with highly conserved stress specific responses such as heat and UV-B. At the same time, our results illustrate the complexity of the stress response that is characteristic of cross-talk pathways [8-14] and multiple secondary effects from prolonged treatments. For example, the early cold stress query signatures (3 h and 6 h) showed very high similarity to cold signatures with only weak similarity scores to other signature phenotypes. Alternatively, the 24 h cold query showed similarity to cold signatures as well as drought and osmotic signatures. This result likely reflects the secondary effects of the prolonged (3 h and 6 h vs. 24 h) cold treatment. Not surprisingly, the co-occurrence of cold and dehydration response reflected in their signaling pathways, or cross-talk, is widely reported in the literature [7,49].

One promising aspect of the signature approach as applied in this study is in the potential use for classification of the dual imposed heat/drought treatment. In nature, a departure from the homeostatic equilibrium, or stress, is often brought about by multiple environmental factors [46]. Heat and drought, for example, are co-occurring stresses that have been implicated in severe yield losses ([46], and citations within). In this study, the highest similarity scores were observed with the heat and drought reference signatures, but the significance of the drought score was dependent upon the depth the signature lists interrogated (see Additional File 5). One explanation for this finding is that the drought responsive transcripts were further down the signature list than the more responsive heat induced transcripts, thereby requiring a greater depth of the signature lists to be compared. This suggests that care must be taken with comparisons between multiple stress phenotypes. However, our results are encouraging in this regard and future research should consider additional statistical means for determining depth of signature list comparisons.

Network theory and analysis was used in an attempt to relate the phenotypic signature information to genome-wide transcriptional programs. Network theory, in general, is promising in this regard because it allows us to view the biology as a system of networks and interacting modules [27]. Here, we use the weighted gene coexpression network approach recently proposed by Zhang and Horvath [20], which has been used successfully to link molecular targets to oncogenic signals [30], complex traits (e.g., mouse weight; [50]), and even network divergence between human and chimpanzee neural patterns [19]. This approach is particularly relevant for our application because it is based on unsupervised clustering, bypasses multiple testing problems when relating gene information to physiological traits, and does not need *a priori *gene ontology information. The latter point is especially important for ecological genomics, which continues to transition from the use of model organisms to those of more ecological relevance.

Results from weighted gene coexpression network analysis produced six distinct modules from the abiotic stress dataset. Importantly, this unsupervised approach grouped genes into network modules that are reflective of biological process. For example, brown module genes clearly participate in photosynthetic processes while turquoise module genes contribute to starch and sucrose regulation. In addition, specific stress responsive modules were identified. The green module, for example, was almost entirely unique to the heat stress pathway and was, in fact, enriched with genes known to participate in heat responsive programs. Equally interesting was the identification of module genes participating in multiple stress responsive pathways. This was apparent for modules consisting of conserved metabolic pathways i.e., brown (photosynthesis) and blue (starch/sucrose metabolism) modules.

One of the more promising aspects of weighted gene coexpression network analysis was the identification of a common abiotic stress responsive module (red module) that enriched differentially expressed genes for all treatments investigated. The most connected gene, or hub, within this module was an uncharacterized ankyrin repeat family protein that was specific to our analysis. Ankyrin proteins have been reported to act as regulators in salicylic acid signaling, which is a key molecule in signal transduction of biotic stress responses [51]. The discovery of this ankyrin family member as the hub in our common stress responsive module suggests that salicylic acid signaling may play a role in abiotic stress response, which would corroborate results from exogenously applied salicylic acid [52]. In addition, this common stress responsive module was enriched with genes known to participate in calcium and calmodulin signaling pathways, which have been shown to participate in a multitude of cellular functions including cell death [53].

Although our findings are robust within the current context, a number of questions remain to be answered. For example, the reference database is generated from immediately perturbed systems that typically exhibit marked and highly significant changes in transcript abundance, and does not include acclimated states where changes in transcript abundance are typically smaller. This has recently been shown in studies investigating changes in gene expression in response to long-term growth at elevated carbon dioxide concentration [54-56]. Therefore the feasibility of scanning the database with a signature from a fully acclimated organism and obtaining a highly correlated signature is uncertain. However, we hypothesize that the acclimated state will also be characterized by unique expression patterns that, in theory, should be amenable to our approach. Like Lamb et al. [22], we are also uncertain how to interpret the significance of the similarity score. Unique to our approach is the use of ordered lists statistics to compare signatures. This statistical test provides a *p*-value based on permuted data that indicates if signature comparisons are more similar than by chance alone. Unfortunately, the interconnectedness among stress responsive pathways resulted in low *p*-values even for some low similarity score comparisons (data not shown). However, we are reluctant to disregard the *p*-value entirely, because as the reference signature database grows and more diverse datasets are included, the *p*-value may help assign phenotypes to general category (e.g., abiotic stress vs. development).

Here, a first-step approach toward classifying and understanding the processes behind the plant stress phenotype is presented. We integrated two analytical techniques that have traditionally been applied only within the biomedical community. Results from our adaptation of the these techniques show that one can take an unknown query signature and through pattern matching software scan a reference database to classify both singular and multiple plant stress phenotype(s). Then, one can use a number of inferential techniques to link phenotypic attributes to their corresponding signaling modules and genes. In essence, this technique provides a tool allowing one to navigate the potential phenotypes of a given *Arabidopsis *genotype. In the current context, the approach is restricted to a single organism. However, a number of technical advances, including sequence-based transcriptomics [57], comparative gene ontology algorithms [58], and analytical approaches for linking network characteristics to quantitative genetics [59] illustrates the potential to enrich our methodology to address questions of evolutionary and ecological interest, particularly physiological trait development.

There are two attributes of our approach that facilitate its use for such purposes. First, the technique is applied within a network framework. Network theory, has been well received in molecular biology for providing a 'systems biology' framework for the discipline (see [60] for a historical perspective), and has more recently been proposed as possible means for determining the evolutionary basis of complex phenotypic traits [61]. Second, and just as important, is the potential to link our approach with population-based genetic analyses. Many of the molecular-based, systems biology experiments are conducted within a narrow adaptive context, with little or no regard for other nonadaptive evolutionary forces (drift, mutation, recombination, gene flow). The inclusion of a genetic association with network analysis, as demonstrated by [59], and placed within a population genetic context allows the appropriate testable null models (e.g., genetic drift) to be included in such studies. Therefore, relating genomic information to genetic information, e.g., quantitative trait loci, is not only possible, but crucial for those interested in exploring the full potential of the evolutionary mechanisms shaping phenotypic development.

Although useful in its current form, we envision that the true potential of our approach will be realized when the scientific community accepts, critiques, and eventually amends our methods with current and future applicable analytical and technological improvements. To facilitate this process, we conducted our analysis within the publicly available R statistical language and make available to the scientific community our signature compendium, R scripts used within, and a brief tutorial illustrating the process, with the near-term goal of providing the community with an integrative systems tool for connecting genes and signaling networks to phenotypic characteristics in order to further the continuing goal of understanding plant genome function.

## Conclusion

In the present study, we detail the initial stages of a theoretical and analytical framework for classifying the plant stress phenotype according to the architecture of the transcriptome, and relate that information to underlying coexpression networks and genes. Our results confirm the existence of known stress-specific genomic signatures, report previously unknown stress-responsive modules and genes, and successfully scale such information to the physiological state of the phenotype. We are encouraged by the results of our present investigation and believe that the approaches developed and information gained here will be critical as we continue to use these tools to better understand species and population-level adaptation to environmental stress, including stress resulting from climate change. Future research in our laboratory is centered on linking functional genomic approaches to genetic information, thereby providing a clear means to pursue evolutionary and ecological genomics at the level of individual organisms, populations, and ecosystems.

## Methods

### Plant growth and treatments

For hydroponic plant production, seeds of *Arabidopsis thaliana *'Columbia' wild-type were cultivated in a modification of the system described in Norén et al. [62]. Briefly, seeds were sterilized in 70% ethanol 5 min, gently agitated in 0.5% SDS solution 15 min, then triple rinsed in sterile Type I water and stratified for 3 d at 4°C. Sterilized seeds were suspended in a 0.1% agarose solution for dispensing onto nutrient agar. Nutrient agar for germination contained 0.5X MS (Sigma-Aldrich) pH 5.7 plus 0.3% phytagar/0.3% phytagel. Approximately 200 μl sterile nutrient agar was placed in the barrel of a 200 μl pipet tip in a 96-well cell well plate. After 2–3 seeds were placed on the cooled and solidified agar, plates were covered with lids, sealed with parafilm, and placed in a CMP 3244-controlled Conviron growth cabinet under 10 h, 22°C days/16 h, 18°C nights with fluorescent lighting (~45 μmol m^-2 ^s^-1^) for 2 weeks until the seedlings had produced 6–8 leaves. The seedlings were then transferred to holes cored into a Styrofoam lid floating atop a 10L plastic tub filled with 1 mM N nutrient solution (1.0 mM KNO_3_, 1.5 mM CaCl_2_, 1.0 mM MgSO_4_·7H_2_0, 1.0 mM K_3_PO_4_·7H_2_0, 0.5 mM NH_4_Cl; micronutrients and Fe-EDTA as described in Norén et al. 2004; and 0.5 gL^-1 ^MES, pH 5.7). Tubs were transferred to a Conviron BWD80-controlled growth room programmed for 10 h, 22°C days/16 h, 18°C nights, 80% RH with aeration provided by aquarium stones attached via tubing to an air pump. Metal halide and tungsten incandescent lamps provided an average of 110 μmol m^-2 ^s^-1 ^within the growth chamber. Seedlings were misted twice daily for approximately one week and lightly covered with a dome to reduce desiccation. A series of shadecloth coverage (70%-50%-30%-no shade) was used to acclimate the plants to MH lights over the course of 7–10 d. During the fourth week the plants were gradually introduced to lower humidity by loosening and finally removing domed lids. After complete chamber acclimation (week 5), plantlets were transferred to JetFlo^® ^Econo Mini hydroponic systems (American Agritech). The system consisted of a 22" × 22" × 7" ebb and flow tray mounted to a 20 gallon reservoir containing 1 mM N nutrient solution as described above with aeration provided by a circulating pump and aquarium stones in both upper and lower chambers.

In total, there were 10 hydroponic systems resulting in a population of over 200 Arabidopsis plants at a relatively mature pre-flowering stage. At the end of 11 weeks, 24 plants were randomly chosen and six each were subjected to the following treatments: (1) Drought- systems drained and roots exposed to air until ~10 % fresh weight had been lost; (2) Heat – systems placed in 38°C chamber for 3 h; (3) plants subjected to combined treatments 1 & 2; and (4) no change (control). At the end of the treatment, plant shoots were harvested and immediately flash frozen in liquid nitrogen for subsequent RNA isolation.

### RNA preparation, design, and microarray hybridization

Total RNA was isolated and labeled from control and treatment shoot tissue using methods from the Vicki Chandler Lab [63]. Briefly, the TRIZOL reagent procedure was initially used to isolate total RNA that was further purified with RNA clean-up spin columns (Qiagen). This two-step process ensured for high quality RNA determined spectrophotometircally (Nanodrop) and with denaturing agarose gels. For each treatment, RNA was transcribed into cDNA primed with T7 Oligo (dT). Amino Allyl-modified RNA was then amplified and purified using manufacturer directions (Ambion) as modified by the Vicki Chandler lab (refer to [63] for protocol). Six μg of each amplified RNA (aRNA) sample was coupled to either CY3 or CY5 monochromeric dye (Amersham) also according to the Chandler protocols. Three μg of labeled aRNA were subsequently used for hybridization onto Arabidopsis slides printed with Operon v 3.1, 70 mer oligos obtained from the laboratory of David Galbraith at the University of Arizona [64]. A formamide-based (50%) hybridization solution was applied at 42°C for 14 h and then washed according to Galbraith protocols [64].

The experiment consisted of one control and three treatments (heat alone, drought alone, heat and drought together) with six replicate plants RNA samples per treatment. To maximize the full power of the design, we used a direct loop design in which half of the samples from each treatment were labeled with CY3 and the other half were labeled with CY5. Therefore, each treatment was directly compared with all other treatments with the possible confounding effects of dye bias controlled for by the inclusion of dye reversals [see Additional file 6].

### Microarray data analysis

Arrays were imaged with a Scan Array 5000 system (Perkin Elmer, Wellesley, MA) and the resultant image files were imported into Imagene v.6.0 for data extraction and initial data diagnostics. All statistical calculations were performed using the limma (v.2.10.5) Bioconductor open access package run in the R statistical framework (R Development Core Team, 2005). Quality of the hybridization was assessed with the Bioconductor packages arrayMagic v.1.14.0 and arrayQuality v.1.12.0. Log-fold (M) over variance (A) plots (M-A plots) were used to determine the most appropriate background correction method. We used basic subtraction in this study, but normexp, normexp offset = 50, and no correction at all were investigated. Arrays were normalized with print-tip loess and no between array normalization was applied. Using limma, a linear model was fitted to compare control to all treatment conditions as contrasts and empirical Bayes was used to compute a moderated t-statistic [25,65]. Multiple testing errors were accounted for by using a false discovery rate (FDR) correction [66].

### Construction of gene coexpression network

Data for network construction came from the AtGenExpress consortium [67] using the first four time-points for cold (4°C), heat (38°C), osmotic (300 mM mannitol), salt (150 mM NaCL), drought (15 min air dry, about 10% loss of fresh weight) and UV-B stresses (15 min exposure, 1.18 w/m^2 ^Phillips TL40W/12). A complete description of experimental design and treatments for the abiotic portion of the AtGenExpress was recently reported by [24]. All hybridizations were performed on the ATH1 affymetrix microarray platform [68]. To reduce noise of the subsequent network, the input gene list was restricted to genes that were differentially expressed at a minimum of one time-point and one treatment. This resulted in a list of 16,036 (~71% of the all non-control array identifiers on the ATH1 slide) unique gene transcript identifiers [see Additional file 1]. Raw intensity values from the gene list were downloaded and log_2 _transformed before being subjected to network construction algorithms.

Construction of the gene coexpression network has been described in detail [20] and R scripts for our network construction were modeled after the Weighted Gene Co-Expression Network [69]. Briefly, the framework for the weighted gene coexpression network (WGCNA) consists of 4 steps: (1) A similarity matrix of gene coexpression is initially determined by the absolute value of the Pearson correlation for all genes across all treatments; (2) transformation of coexpression similarities into connection strengths (connectivity) using a power adjacency function; (3) identification of network modules (highly correlated gene expression patterns across samples) by coupling linkage hierarchical clustering with topological overlap matrix; and (4) relating external gene or sample information to network properties. Details and tutorials of the WGCNA are available at the website.

### Signature comparisons

Similar to Lamb et al. [22], the first step in our process was to create a catalog of reference signatures representing known biological states, plant stress phenotypes in our case. As a first step in this process, raw .cel files from the AtGenExpress dataset were downloaded and analyzed for differential expression. We again used the limma (v.2.10.5) Bioconductor open access package run in the R statistical framework. The entire dataset was normalized using gcRMA procedures and the linear model was fitted and contrasts were restricted to within treatment conditions. A moderated t-statistic was computed using empirical Bayes [25,65] and a FDR correction [66]. The log-fold change in expression relative to the control was used to rank the lists. Therefore, the first gene on the list represented the most over-expressed gene in relation to the control while the last gene in the signature represented the most down-regulated gene in relation to the control.

The reference signature database therefore consisted of six treatments for the first four time-points, resulting in 24 independent signatures. The rank-based nature of the signatures allowed us to use list statistical tests from the OrderedLists Bioconductor statistical package [43,44]. The test includes a user defined value for how many genes to consider in the list comparison. In our case, we found that an alpha level of 0.3 interrogated a substantial portion of the list while focusing on list portions that were significantly differentially expressed for all conditions. The similarity score is computed as the shared number of genes between both signatures (lists) including the weighted sum score as determined by a weighting vector (refer to [27]). Therefore, the ends of the list are weighted most highly and contribute more to the similarity score, ensuring that the most differentially expressed genes contribute most to the score. Similar to Lamb et al. [22], the output consists of a non-parametric similarity score generated from the number and weights of the rank-based comparison between query and reference signatures. In addition, a *p-*value is calculated based on the rank of the query signature genes compared to the rank of the reference profile genes and a 1000 random permutations of the reference profile (refer to [43]). Therefore, a significant *p*-value means that the genes in common to the lists are not due to chance.

### Availability of the methods and data

As mentioned in the manuscript, we present only a prototype version with limited number of signature phenotypes represented. The full potential of this resource, however, will become apparent when the representative phenotypes grow and began to exhaust that realized by the genome, and eventually genomes from other ecotypes and closely related species. Therefore, our microarray analysis has been deposited in the Gene Omnibus database (GEO: GSE9415). In addition, a the R scripts to reproduce our weighted gene coexpression network results [see Additional file 7] as well as the complete file of all reference genomic signature lists has been provided [see Additional file 8]. Information regarding network theory and R-code tutorials for weighted gene coexpression networks is available from the Steve Horvath Lab [69].

## Authors' contributions

DJW formulated the experimental questions and design, performed all analyses, and drafted the manuscript. LEG was responsible for plant propagation, assisted in experimental treatment and microarray hybridizations, and helped to draft the manuscript. AR critically annotated gene lists and helped draft the manuscript. SDW assisted with experimental design, data interpretation, and helped draft the manuscript. All authors read and approved the final manuscript.

## Supplementary Material

Additional file 1 Supplementary Table 1; Gene network coexpression gene input list. Click here for file

Additional file 2 Supplementary Table 2; Module gene enrichment of differentially expressed genes. Click here for file

Additional file 3 Supplementary Table 3; Results for the weighted gene coexpression network analysis. Click here for file

Additional file 4 Supplementary Table 4; Signature intersect gene annotation and network properties. Click here for file

Additional file 5 Supplementary Figure 1; Similarity score comparison of individual replicates of the combined heat and drought treatment. Click here for file

Additional file 6 Supplementary Figure 2; Microarray hybridization design schematic. Click here for file

Additional file 7 R scripts used to construct the weighted gene coexpression network. Click here for file

Additional file 8 The reference genomic signatures lists. Click here for file
