# Supplementary material for: Connecting genes, coexpression modules, and molecular signatures to environmental stress phenotypes in plants
Source: BMC Syst Biol. 2008 Feb 4;2:16. doi: 10.1186/1752-0509-2-16 (PMC2277374; doi:10.1186/1752-0509-2-16)

Supplementary Figure 1.

The ranked-based nature of the genomic signatures allowed us to use the OrderedLists bioconductor package to find similar genes based upon their rankings between query and reference signatures. One of the ambiguities in comparing lists, such as our signatures, is in defining the number of genes (or elements) to consider in the list comparisons (refer to materials and methods). Throughout the manuscript, we used an alpha level of 0.3 which defines the depth of the list comparisons. This interrogated a substantial portion of the signature while focusing on genes that were significantly differentially expressed for all conditions. However, when investigating the simultaneously imposed heat and drought query signature against the compendium, both heat and drought had high similarity scores but the significance of the scores was difficult to access. To gain further insight into similarity score comparisons, we scanned the six independent query replicates against the compendium. Again, drought and heat were the highest similarity scores yet the drought score was not significantly differently from some of the non-heat signatures (see Fig. A). If we change the alpha level to 1, which compares a greater portion of the signature genes, then the drought score begins to separate from the other non-heat scores (Fig. B). This is likely due to the dominance of heat related transcripts in the upper-most portion of the lists (see Fig. 6), which drives the drought responsive transcripts further down the signature list. This illustrates the need to understand the hierarchy of module gene enrichment of differentially expressed genes when comparing similarities between signature lists.

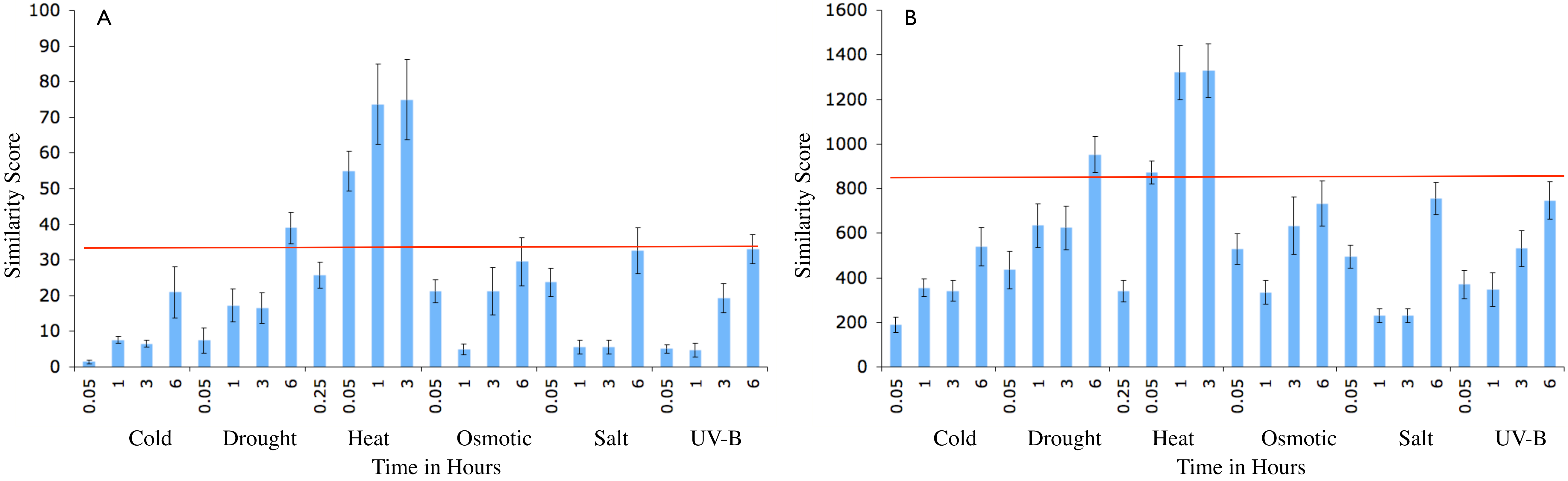

Supplement: Additional file 5 — Supplementary Figure 1; Similarity score comparison of individual replicates of the combined heat and drought treatment. [file 1752-0509-2-16-S5.PDF]
