# Supplementary material for: Connecting genes, coexpression modules, and molecular signatures to environmental stress phenotypes in plants
Source: BMC Syst Biol. 2008 Feb 4;2:16. doi: 10.1186/1752-0509-2-16 (PMC2277374; doi:10.1186/1752-0509-2-16)
Supplement: Additional file 6 — Supplementary Figure 2; Microarray hybridization design schematic. [file 1752-0509-2-16-S6.PDF]

Supplementary Figure 2. Microarray hybridization design. The tail of the arrow is the Cy3 dye and the head of the arrow is the Cy5 dye. One arrow represents a single hybridization and array slide. Array data is available on the GEO database.

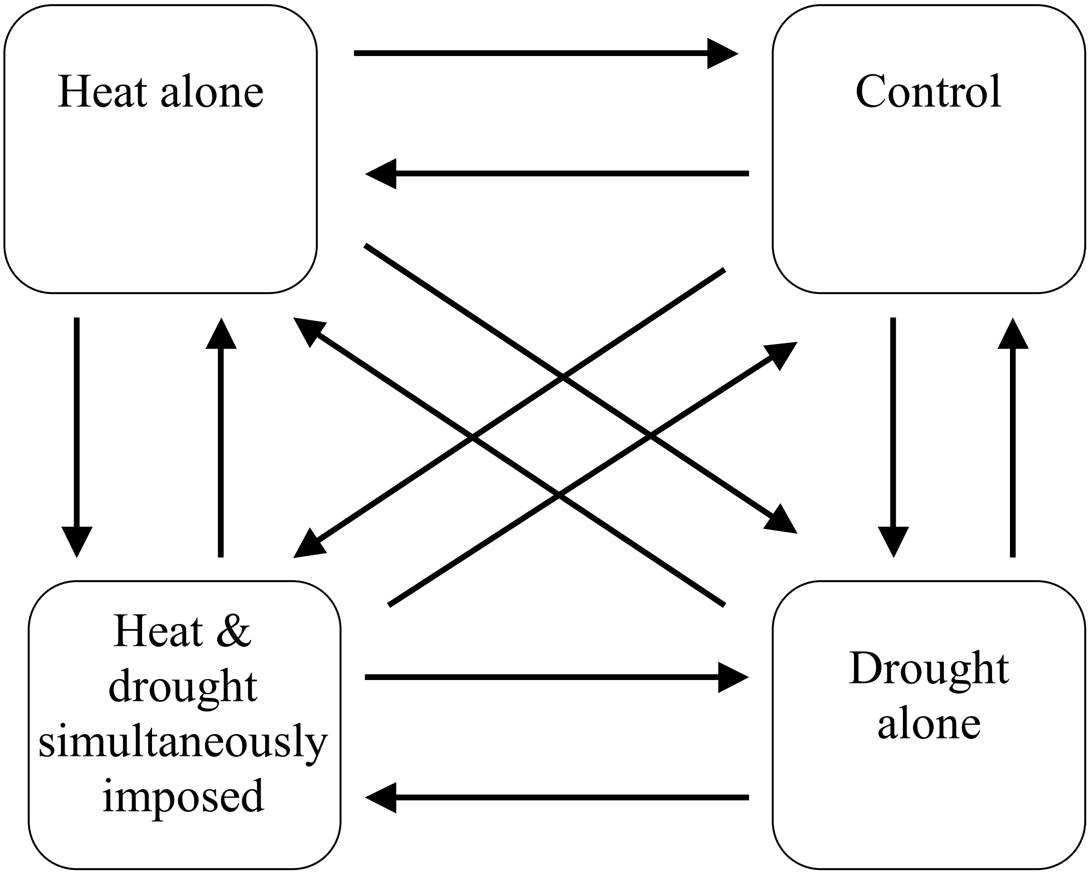

Name	FileNameCy3	FileNameCy5	Cy3	Cy5
hyb1	slide1_Cy3.txt	slide1_Cy5.txt	Heat alone	Control
hyb2	slide2_Cy3.txt	slide2_Cy5.txt	Control	Heat alone
hyb3	slide3_Cy3.txt	slide3_Cy5.txt	Drought alone	Control
hyb4	slide4_Cy3.txt	slide4_Cy5.txt	Control	Drought alone
hyb5	slide5_Cy3.txt	slide5_Cy5.txt	Control	Drought and Heat
hyb6	slide6_Cy3.txt	slide6_Cy5.txt	Drought and Heat	Control
hyb7	slide7_Cy3.txt	slide7_Cy5.txt	Drought alone	Heat alone
hyb8	slide8_Cy3.txt	slide8_Cy5.txt	Heat alone	Drought alone
hyb9	slide9_Cy3.txt	slide9_Cy5.txt	Drought and Heat	Heat alone
hyb10	slide10_Cy3.txt	slide10_Cy5.txt	Heat alone	Drought and Heat
hyb11	slide11_Cy3.txt	slide11_Cy5.txt	Drought alone	Drought and Heat
hyb12	slide12_Cy3.txt	slide12_Cy5.txt	Drought and Heat	Drought alone
