# Supplementary material for: Connecting genes, coexpression modules, and molecular signatures to environmental stress phenotypes in plants
Source: BMC Syst Biol. 2008 Feb 4;2:16. doi: 10.1186/1752-0509-2-16 (PMC2277374; doi:10.1186/1752-0509-2-16)
Supplement: Additional file 7 — R scripts used to construct the weighted gene coexpression network. [file 1752-0509-2-16-S7.DOC]

Additional File 7. R scripts to reproduce the results from our weighted gene coexpression network analysis.

Construction of the gene coexpression networks has been described in detail previously from the Horvath Lab and R scripts for our network construction were modeled after the Weighted Gene Co-Expression Network ([www.genetics.ucla.edu/labs/horvath/ CoexpressionNetwork/](http://www.genetics.ucla.edu/labs/horvath/ CoexpressionNetwork/)). The R scripts provided here are simply given so that one can reproduce and further investigate the results reported within this manuscript. For complete tutorials on weighted gene coexpression network analysis you must visit the Horvath Lab website.

To begin, you need to download the latest version of R from <http://www.r-project.org/>, the latest networkfunctions.txt file from the Horvath website above, and the R library packages from CRAN.

#Copy and paste the following scripts into the R command window. If you are missing #any of these packages, download them from your CRAN mirror described in #<http://www.r-project.org/>.

library(MASS)

library(class)

library(cluster)

library(sma)

library(impute)

library(scatterplot3d)

library(Hmisc)

# Choose your working directory from which all relevant files are located, including the #NetworkFucntions.txt from the Horvath website and the network_stress_input.txt, which #is provided as Additional File 1 on the BMC Systems biology website. Note that #Additional File 1 needs to downloaded, opened in Excel, and saved as #network_stress_input.txt in the chosen working directory.

source("NetworkFunctions-1.txt")

dat0<-read.table("network_stress_input.txt", header=TRUE, sep="\t")

names(dat0)

datSummary<-dat0[,c(1:1)]

datExpr<-t(dat0[,2:65])

dim(datExpr)

rm(dat0)

collect_garbage()

#to pick a powers threshold____________________________

powers1=c(seq(1,10,by=1), seq(12,20, by=2))

RpowerTable=PickSoftThreshold(datExpr,powervector=powers1)[[2]]

collect_garbage()

cex1=0.7

par(mfrow=c(1,2))

plot(RpowerTable[,1], -sign(RpowerTable[,3])*RpowerTable[,2],xlab="

Soft Threshold (power)",ylab="Scale Free Topology Model Fit,signed R^2",type="n")

text(RpowerTable[,1], -sign(RpowerTable[,3])*RpowerTable[,2], labels=powers1,cex=cex1,col="red")

abline(h=0.8,col="red")

plot(RpowerTable[,1], RpowerTable[,5],xlab="Soft Threshold (power)",ylab="Mean Connectivity", type="n")

text(RpowerTable[,1], RpowerTable[,5], labels=powers1, cex=cex1,col="red")

#here we chose beta1=10

#Note that we are using signed functions so that genes within module are

#positively correlated.

beta1=10

DegreeSoft=SoftConnectivity(datExpr,power=beta1)-1

Degree=DegreeSoft

DegCut=4000 #this is the number of genes

DegreeRank=rank(-Degree)

restDegree=DegreeRank<=DegCut

sum(restDegree) ## we had null here

CORO=cor(datExpr[,restDegree],use="p")

ADJ=((CORO+1)/2)^10

dissGTOMsignedPower10=TOMdist1(ADJ)

hierGTOMsignedPower10=hclust(as.dist(dissGTOMsignedPower10),method="average");

par(mfrow=c(2,1))

plot(hierGTOMsignedPower10,labels=F)

colorhsignedPower10versionB=as.character(modulecolor2(hierGTOMsignedPower10,h1=.9,minsize1=50))

table(colorhsignedPower10versionB)

hclustplot1(hierGTOMsignedPower10, colorhsignedPower10versionB, title1="Colored by SIGNED modules, power=10")

summary(colorhsignedPower10versionB)

collect_garbage()

#TOM plot

TOMplot1(dissGTOMsignedPower10, hierGTOMsignedPower10, colorhsignedPower10versionB)

#This should reproduce the results of the weighted gene coexpression network. For #additional information and diagnostics, refer to the Horvath Lab website as mentioned #above.
